# Estrus response and pregnancy rate of swamp buffalo: The use of multivitamins in different estrus synchronization hormone protocols

**DOI:** 10.5455/javar.2024.k824

**Published:** 2024-09-29

**Authors:** Ferry Lismanto Syaiful, Jaswandi Jaswandi, Mangku Mundana, Yendraliza Yendraliza, Zaituni Udin

**Affiliations:** 1 Department of Animal Production Technology, Faculty of Animal Science, Universitas Andalas, Padang, Indonesia; 2 Faculty of Agriculture and Animal Science, Universitas Islam Negeri Sultan Syarif Kasim Riau, Pekanbaru, Indonesia

**Keywords:** Estrus synchronization, hormone synchronization protocol, multivitamin, estrus response, pregnancy rate, swamp buffalo

## Abstract

**Objective:**

This study aims to determine the effect of using multivitamins in different estrus synchronization hormone protocols on estrus response, estrus onset, estrus duration, estrus intensity, and pregnancy rate in swamp buffalo.

**Materials and Methods:**

This study used 30 post-partum adult buffalo, with three estrus synchronization methods treated: 1. Conventional plus Prostaglandin F2 α (PGF2α-PGF2α + multivitamin)-AI; 2. Co-synch plus Gonadotropin-releasing hormone (GnRH-PGF2α + multivitamin)-AI; 3. Combination of hormone plus (Estrogen-Progesterone-PGF2α + multivitamin)-AI. Research variables include estrus response, estrus onset, estrus duration, estrus intensity, and pregnancy rate. Data were analyzed using the chi-square test using the Statistical Package for the Social Sciences 23.0 program.

**Results:**

The results showed that the use of multivitamins in different estrus synchronization hormone protocols resulted in an estrous response reaching 100%. The onset of estrus in the three treatments [Treatment-1 (T1); Treatment-2 (T2); Treatment-3 (T3)] was 25.8; 27.6; 23.9 h, estrus duration: 21.0; 21.6; 21.92 h, estrus intensity: 25.8; 27.6; 32.6 h, and the pregnancy rate for buffalo reaches 80%.

**Conclusion:**

Based on the results of this study, the use of multivitamins in different estrus synchronization hormone protocols is effective in optimizing the swamp buffalo’s estrus response; the estrus duration is longer, the estrus onset is faster, and the estrus intensity is higher. It can even optimize the increase in swamp buffalo pregnancy rates.

## Introduction

Buffalo (*Bubalus bubalis*) is a ruminant livestock producing meat and milk, which can make a major contribution to realizing food self-sufficiency in Indonesia. These livestock also play an important role in agriculture and community life. However, in recent years, the buffalo population has experienced a significant decline. This decline was caused by low productivity because buffalo have several characteristics, including silent heat, low estrus intensity, and short estrus duration, resulting in longer calving intervals and a decrease in the birth rate of buffalo calves. According to Pirondi et al. [1] and Purohit et al. [2], buffalo have the characteristics of silent heat, low intensity of estrus, and short duration of estrus, which causes a decrease in reproductive efficiency and a decline in the buffalo population. It is necessary to identify problems and find appropriate solutions to increase the productivity and population of buffalo through livestock biotechnology (estrus synchronization and artificial insemination/AI) and the use of multivitamins and nutrition. Implementing this step is expected to increase the reproductive efficiency and productivity of buffalo to improve the birth of buffalo calves. According to Ahmad and Arshad’s research [3], Carvalho et al. [4], and De Rensis and López-Gatius [5], the reproductive efficiency of buffalo can be optimized through various hormonal approaches, such as estrus synchronization techniques.

Estrus synchronization is a technical method used to control the estrus cycle of a group of livestock, leading to simultaneous estrus. This technique involves the use of a specific hormone protocol inducing estrus in livestock to facilitate simultaneous insemination. Estrus synchronization is an important strategy for increasing the reproductive efficiency and fertility of buffalo. Several estrus synchronization methods have been used: Ovsynch [6,7], Co-synch [7,8], and conventional methods [5]. According to Warriach et al. [9] and Lauderdale [10], buffalo estrus synchronization protocols generally involve the use of hormones such as Prostaglandin F2 α (PGF2α), Gonadotropin-releasing hormone (GnRH), and progesterone (P4), as previously reported by Haider et al. [11] and Naseer et al. [12]. These protocols have the potential to increase livestock fertility. According to Efendi et al. [7], the use of estrus synchronization hormones such as PGF2α intramuscularly and other methods (ovsynch and co-synch) have obtained effective results. According to Yendraliza et al. [13], combining GnRH and PGF2α hormone protocols in post-partum buffaloes can increase estrus intensity, accelerate estrus response, and reduce estrus duration. Furthermore, Yousuf et al. [14] suggested that the combined use of the hormone progesterone and estradiol benzoate can stimulate follicular waves and increase the rate of follicular growth and the intensity of estrus. Confirmed by Campanile et al. [15] that the use of various estrus synchronization hormone protocols can help the formation of the corpus luteum and increase the concentration of progesterone, thus helping embryo implantation and placental development in supporting the survival of the embryo. According to Purohit et al. [2] and De Rensis and López-Gatius [5], the use of estrus synchronization protocols can increase livestock reproductive efficiency, estrus response, and pregnancy rate of female buffalo.

On the other hand, Chadda and Chand Meena [16] suggested that lack of livestock nutrition and vitamins can interfere with follicle maturation, causing a long livestock estrous cycle. Hafez and Hafez [17] explain that vitamin deficiencies can cause estrus and ovulation disorders, causing the production of a low number of livestock eggs. According to Sarker et al. [18], the administration of vitamins in the synchronized GnRH hormone protocol can increase the pregnancy rate of cattle. According to Likittrakulwong et al. [19], administering vitamin AD_3_E can increase antioxidants and improve the immune system of livestock. According to Tamura et al. [20], during the ovulation process of livestock, free radicals are often produced, causing oxidative stress, which decreases oocyte quality, even infertility, and impaired embryonic development. Dunne et al. [21] stated that the vitamins AD_3_E and C were the antioxidants that could neutralize free radicals to prevent infertility and embryo death. Vitamin A is very useful for vision, bone growth, and immune function [22]. Vitamin D_3_ can influence the estrus cycle of post-partum cattle and calving interval [23]. Vitamin E plays a role in protecting cellular lipids from free radical oxidation, which can damage cellular metabolism [8]. Vitamin C can eliminate free radicals and activate vitamin E [24]. Therefore, the author is interested in researching the effect of using multivitamins in various estrus synchronization hormone protocols in swamp buffalo.

## Materials and Methods

### Ethical approval

Experimental research on swamp buffalo has been evaluated and approved by the Research Ethics Committee, Faculty of Medicine, Universitas Andalas. No. 408/UN.16.2/KEP-KP/2024.

### Research material

This research material used 30 post-partum female swamp buffalo from Batang Anai District, Padang Pariaman Regency, West Sumatra, Indonesia. The chemicals used in this research were the PGF2α hormone (Lutalyse), the GnRH hormone (Fertagyl^®^, Intervet, Indonesia), vitamin ADE, Vigatol-E (Bayer), gel, NaCl, phosphate buffer saline, and 70% alcohol. The equipment used is gloves, scissors, tweezers, aluminum foil, label paper, tissue, and AI equipment.

### Collection and selection of research buffalo

The method used in this research was purposive sampling (according to criteria). Criteria for selecting livestock include post-partum adult buffalo, parity 2–3, Body condition score ≥3, healthy livestock, and not pregnant. Furthermore, the buffalo selected were buffalo that were reared semi-intensively (grazing from morning to evening, being penned at night, and having access to drinking water ad libitum).

### Estrus synchronization

**Figure 1.**
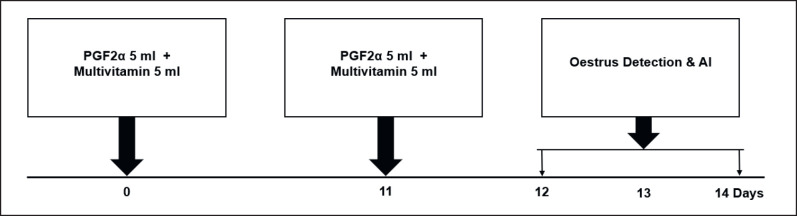
Conventional plus method (PGF2α-PGF2 α + Multivitamin)—AI.

**Figure 2.**
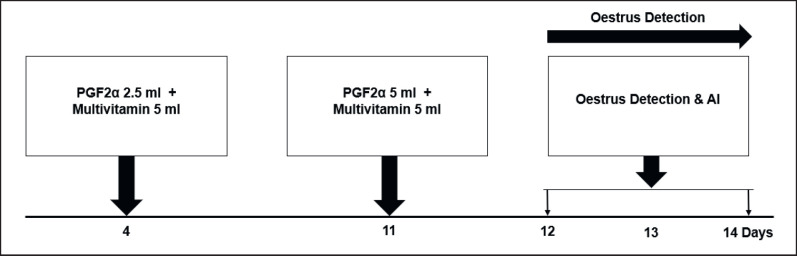
Co-synch plus method (GnRH-PgF2α + Multivitamin)AI.

**Figure 3.**
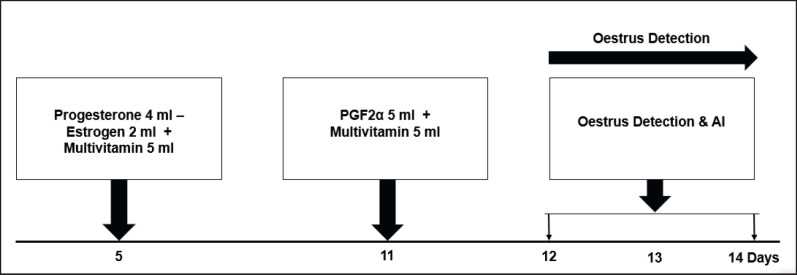
Combination hormone plus method (Estrogen-Progesterone-PGF2α + Multivitamin)—AI.

This research used three treatments, and each treatment used 10 swamp buffalo. The research treatments were: Treatment-1 (T1): conventional plus (PGF2α-PGF2α + Multivitamin-AI); Treatment-2 (T2): co-synch Plus (GnRH-PGF2α + Multivitamin-AI); Treatment-3 (T3): combination of hormones plus (Estrogen-Progesterone-PGF2α + Multivitamin-AI). Hormone combination plus (T3) includes estrogen-progesterone-PGF2α + Multivitamin-AI. This research procedure refers to the research of Yendraliza et al. [13], Atabay et al. [25], Afriani et al. [26], and Susana et al. [27], which has been modified. For the T1, estrus synchronization was carried out using the conventional plus method (PGF2α-PGF2α + 10 ml multivitamin). Each treatment was injected twice intramuscularly into the treated buffalo. The first injection was given on day 0 (PGF2α 5 ml), followed by administration of 5 ml of multivitamin. Then, the second injection was carried out on day 11 (PGF2α 5 ml), followed by a multivitamin injection of 5 ml. Furthermore, estrus detection is carried out on days 12 to 14. If the buffalo shows symptoms of estrus, artificial insemination (AI) is carried out, and the implementation procedure is described in Figure 1. T2: Synchronization was carried out using the co-synch plus method (GnRH-PGF2α-GnRH + 10 ml multivitamin). Each treatment was injected twice intramuscularly into the treated buffalo. The first hormone injection was given on day 4 (GnRH 2.5 ml), followed by administration of 5 ml of multivitamin. Then, the second hormone injection (PGF2α 5 ml) was followed by the administration of 5 ml of multivitamin, which was carried out on the 11th day. On days 12–14, estrus is observed. If the buffalo shows signs of estrus, then AI is carried out, and the implementation procedure is described in Figure 2. In the T3, estrus synchronization was carried out using a combination of the hormones estrogen and progesterone (estrogen-progesterone-PGF2α + 10 ml multivitamin). Each treatment was injected twice intramuscularly into the treated buffalo. The first hormone injection is given on day 5 (4 ml progesterone hormone + 2 ml estrogen), followed by administration of 5 ml multivitamin. Then, the second hormone injection (PGF2α 5 ml) followed by administration of 5 ml of multivitamins was carried out on the 11th day. On days 12–14, estrus is observed. If the buffalo shows signs of estrus, then AI is carried out, and the implementation procedure is shown in Figure 3.

### AI and pregnancy detection in buffalo

If buffalo show signs of estrus, AI is carried out using frozen semen at the Centre for AI of Lembang. After insemination, a pregnancy check is carried out on the 90th day using rectal palpation. The research variables are estrus response, estrus onset, estrus duration, estrus intensity, and pregnancy rate. Data analysis was carried out using the chi-Square test using the Statistical Package for the Social Sciences 23.0 program.

## Results and Discussion

### Estrus response

The estrus response of swamp buffalo with the use of multivitamins in various estrus synchronization hormone protocols is described in Table 1. Table 1 shows that the use of multivitamins in various estrus synchronization hormone protocols can increase the estrus response by up to 100%. Statistical analysis showed that the three estrus synchronization methods being used had no significant effect on the estrous response (*p* > 0.05). The results of this research prove that the use of multivitamins in various estrus synchronization hormone protocols is effective in increasing the reproductive cycle of buffalo and can optimize the estrus response. According to Intawicha et al. [28], the use of a synchronization hormone protocol can improve the estrous response of post-partum buffalo because hormone administration can stimulate ovarian activity to trigger an increase in the estrus response. According to Farrag [29] and Kuru et al. [30], the use of an estrus synchronization hormone protocol can improve the estrus response, and ovulation induction is even more effective in optimizing the increase in the pregnancy rate of livestock. The results of this research are better than the research done by Suzana et al. [27], Atabay et al. [25], Yendraliza et al. [31], and Roza et al. [32]. Their research results only reached an estrus response rate of 70%–97%. The high results obtained in this study were due to the use of multivitamins in various estrus synchronization hormone protocols. The use of multivitamins can improve the reproductive function and structure of livestock. According to Prasdini et al. [33] and Yosathai [34], the use of vitamins, especially vitamins AD_3_ and E, can improve the reproductive function and structure of livestock. Vitamin E acts as an antioxidant, stimulating the processes of steroidogenesis, thyroid hormone secretion, and follicle development for ovulation. Meanwhile, vitamin ADE can increase livestock fertility. Confirmed by Purwasih et al. [35] and Dewi et al. [36] that the use of vitamins AD_3_E and GnRH can overcome reproductive disorders, recurrent mating problems, and even increase ovulation in livestock. As supported by Gunawan et al. [37], using the estrus synchronization hormone protocol twice can increase the buffalo’s estrus response optimally. The results of this study show that the use of multivitamins in different estrus synchronization protocols can optimally increase the estrous response of post-partum buffalo up to 100%. The high level of estrus response can increase reproductive efficiency and livestock productivity, which improves the pregnancy rate of buffalo.

**Table 1.** Estrus response of swamp buffaloes to multivitamin supplementation in different estrus synchronization hormone protocols.

No	Synchronization of Estrus Method	n (Tail)	The number of livestock in estrus (Tail)	The number of livestock not in estrus (Tail)	Estrus Percentage (%)	Pregnancy Percentage (%)
1	Conventional method plus multivitamin (PGF2α-PGF2α + Multivitamin 10 ml)	10	10	0	100	80
2	co-synch method plus multivitamin (GnRH-PGF2α + Multivitamin 10 ml)	10	10	0	100	80
3	Hormone Combinations method plus multivitamin (Estrogen 2 ml-Progesterone 4 ml-PGF2α 5 ml + Multivitamin 10 ml)	10	10	0	100	80
Total		30				

### Onset of estrus

**Figure 4.**
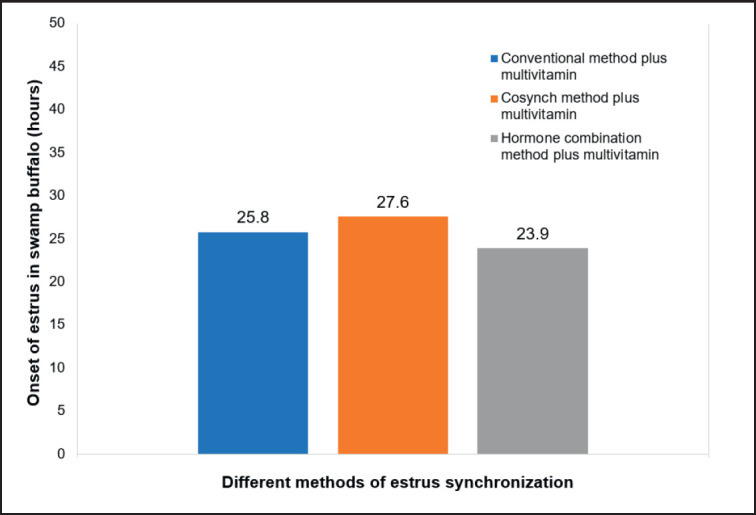
The onset of estrus in swamp buffalo with the use of multivitamins in different estrus synchronization hormone protocols.

The onset of estrus in swamp buffalo with the use of multivitamins in various different estrus synchronization hormone protocols is described in Figure 4. Figure 4 shows that the highest onset of buffalo estrus was achieved at T2 (co-synch + multivitamin), namely 27.6 ± 2.26 h, while the lowest was obtained at T3 (combination of estrogen and progesterone hormones + multivitamin) at 23.90 ± 2.18 h. Statistical analysis showed that the use of multivitamins in different estrus synchronization hormone protocols had no significant effect on the onset of estrus in buffalo (*p* > 0.05). This is because the use of multivitamins in different estrus synchronization hormone protocols has the potential to increase follicle development and estrus expression. Purohit et al. [2] suggested that the use of multivitamins can improve the quality and quantity of buffalo ovarian follicles, thereby increasing the estrus response. It was confirmed by Zhao et al. [38] that hormone administration can accelerate the onset of estrus, increase the production of the hormone estradiol, and affect the development of ovarian follicles. According to Koyama et al. [39], the onset of estrus can function as a predictive factor for the timing of ovulation and can even increase the pregnancy rate of cattle. The results of this research are superior to the research of Yendraliza et al. [13] that using the GnRH-PGF2α hormone protocol obtained an estrus onset of 30.80 h, which was faster than the onset of estrus using the PGF2α-PGF2α hormone, which was obtained at 39.05 h. According to Susana Koyama et al. [39], the use of an estrus synchronization protocol can result in an estrus onset between 22.45 and 31.02 h. Meanwhile, according to Afriani et al. [26], administering the GnRH hormone to synchronize buffalo estrus, estrus onset was achieved in 18.2 h. This difference is caused by livestock condition, age, season, feed nutrition, method, and estrus synchronization hormone protocol. According to Dewi et al. [36], the effectiveness of estrus synchronization protocols is influenced by the health and physiological conditions of livestock, both of which can influence ovarian activity. According to Perera [40], the use of the GnRH-PGF2a hormone can accelerate the onset of estrus in buffalo and facilitate follicular growth for corpus luteum proliferation. Supported by Efendi et al. [7] that the combination of hormones (GnRH-PGF2a) (Co-synch) is better than the conventional method (PGF2α-PGF2α). It was confirmed by Gordon [41] that the use of the GnRH hormone in post-partum buffalo can increase the ovarian cycle, improve corpus luteum disorders, and reduce the hormone progesterone.

**Figure 5.**
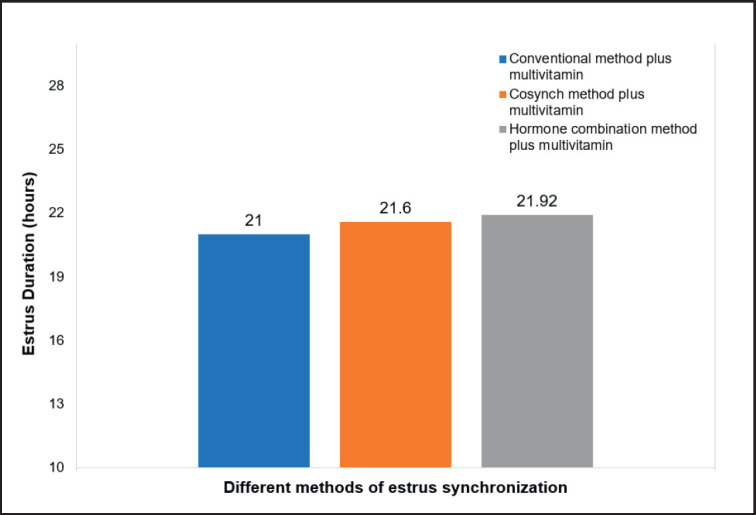
The estrus duration of swamp buffaloes with the use of multivitamins in different estrus synchronization hormone protocols.

This result shows that the use of multivitamins in the GnRH-PGF2a hormone protocol can accelerate the onset of buffalo estrus. According to Setyorini and Prihatno [42], the administration of multivitamins with the GnRH hormone is effective in overcoming repeated mating and increasing pregnancy rates in livestock. The onset of estrus is important in the reproductive management of buffalo and can even obtain the ideal time expected for insemination. Moreover, the use of multivitamins in different estrus synchronization protocols is very good in supporting the acceleration of estrus onset to achieve optimal estrus response and even has the potential to increase the pregnancy rate of the buffalo.

### Duration of estrus

The duration of swamp buffalo estrus with the use of multivitamins in various estrus synchronization hormone protocols is described in Figure 5. Figure 5 shows that the highest estrus duration was obtained at T3 (hormone combination + multivitamin method) in 21.92 h, while the lowest estrus duration was obtained at T1 (conventional method + multivitamin) in 21 h. The absence of this difference is due to the hormonal interactions being used (synchronizing hormones and multivitamin supplementation), which can improve livestock health and reproductive efficiency. Therefore, it does not affect the duration of estrus. According to Chadda and Meena [16], the balance between hypostasis hormones and LH can produce the same estrus duration in livestock. Supported by Purohit et al. [2], Kuru et al. [43], and Chaudhari et al. [44], the duration of buffalo estrus is affected by hormonal status, and the administration of multivitamins indirectly impacts hormonal levels. According to Hafez and Hafez [17], the use of vitamin ADE can overcome disturbances in estrus. Administration of multivitamins can indirectly influence the duration of estrus by increasing the quality and quantity of ovarian follicles so that the onset of estrus is faster and the duration of estrus is shorter.

**Table 2.** Estrus intensity of synchronized water buffaloes using various estrus synchronization methods (h).

No	Synchronization of Estrus Method	n (Tail)	Score	Estrus intensity (h)	Category of estrus intensity
1	Conventional method plus multivitamin (PGF2α-PGF2α + Multivitamin 10 ml)	10	+++	25.80^a^ ± 2.74	High
2	Co-synch method plus multivitamin (GnRH-PGF2α + Multivitamin 10 ml)	10	+++	27.60^ab^ ± 2.26	High
3	Hormone Combinations method plus multivitamin (Estrogen 2 ml-Progesterone 4 ml-PGF2α 5 ml + Multivitamin 10 ml)	10	+++	32.60^bc^ ± 3.19	High

**Note**: Data is presented in numbers and standard deviation (SD).

The results of this study are by the research by Perera [40]. The duration of buffalo estrus ranges from 5 to 27 h. However, the results of this study are different from Yendraliza et al. [31], where the duration of buffalo estrus ranged from 6.5 to 18.6 h. Meanwhile, Afriani et al. [26] explained that the duration of estrus using GnRH was 18 h. This difference is caused by the health condition of the livestock, the type of livestock, and the hormone supplementation used. According to Irmaylin and Madi [45], the duration of cattle estrus is affected by the age of the cattle, their condition and health, estrus induction, and the hormones used. Supported by Shahid et al. [46], the duration of estrus can increase estrus induction, which causes the estrus duration of livestock to be longer. Therefore, it is beneficial for AI programs and the genetic improvement of livestock. The use of multivitamins in various estrus synchronization protocols is suitable for optimizing buffalo estrus synchronization.

### Estrus intensity

The intensity of swamp buffalo estrus with the use of multivitamins in various estrus synchronization hormone protocols is described in Table 2. Table 2 shows that there are differences in the estrus intensity of swamp buffaloes synchronized using different estrus synchronization methods. The highest duration was obtained at T3 (the combination method of estrogen and progesterone hormones plus multivitamins) at 32.60 h, while the lowest was at T1 (conventional method plus multivitamins) at 25.80 h. The results of this study show that using the hormones estrogen and progesterone can increase the intensity of buffalo estrus compared to other treatments. The use of multivitamins in various estrus synchronization hormone protocols obtained high estrus intensity values ( + + + ). Supported by Ramli et al. [47], a high estrus score ( + + + ) has the criterion that the signs of estrus are clearer, indicating the optimal period for insemination. In addition, high estrus intensity can increase animal conception and reduce repeated mating, which improves reproductive efficiency and animal productivity.

**Figure 6.**
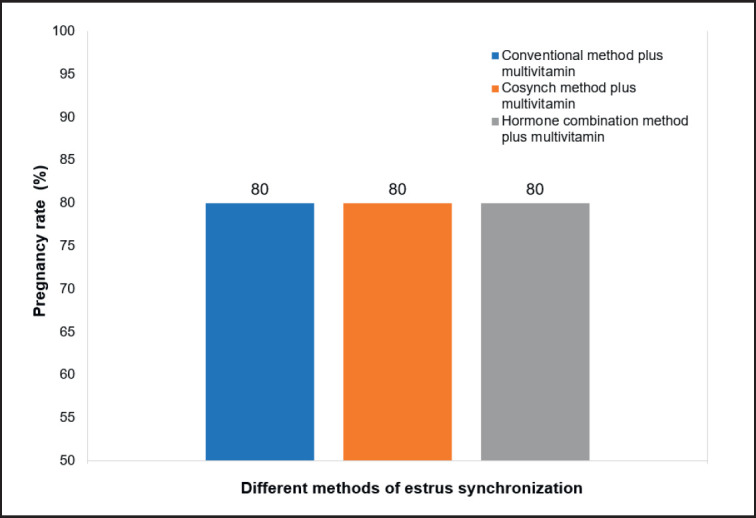
The pregnancy rate of swamp buffalo with the use of multivitamins in various estrus synchronization hormone protocols.

### Pregnancy rate

The pregnancy rate of swamp buffalo with the use of multivitamins in various estrus synchronization hormone protocols is described in Figure 6. Figure 6 shows that the use of multivitamins in various estrus synchronization hormone protocols produced a pregnancy rate for buffalo of 80%. The results of this study are by Syaiful et al. [48] that the accuracy of detecting buffalo pregnancy using rectal palpation is 80%, and using ultrasound can reach 100%. This result also proves that the use of multivitamins in different hormone protocols can increase the fertility of buffalo and reduce embryo mortality to optimize the increase in buffalo pregnancy rates. According to Purohit et al. [2] and De Rensis and Gatius [5], the use of different estrus synchronization hormone protocols is effective in increasing the fertility level of buffalo. According to Sanker et al. [49], there are several factors affecting livestock fertility, such as livestock health conditions, hormone synchronization protocols, and livestock reproductive management. As confirmed by Nava-Trujillo et al. [50] and Ahmad and Arshad [3], the fertility level of livestock is affected by the response of livestock to estrus synchronization and AI treatments. Furthermore, Oliveira Filho et al. [51] stated that the estrus response can increase livestock fertility. Supported by Syaiful et al. [52], livestock fertility levels are also affected by genetics, animal age, parity, health conditions, and feed management. Furthermore, Oliveira Filho et al. [53] suggested that the reproductive efficiency of buffalo can be increased through estrus synchronization methods. The use of various estrus synchronization hormone protocols can help the formation of the corpus luteum and increase the concentration of progesterone, thus helping embryo implantation and placental development in supporting embryo survival to increase the pregnancy rate of buffalo.

## Conclusion

Based on the results of this research, using multivitamins in various estrus synchronization hormone protocols effectively optimizes the buffalo’s estrus response, reaching up to 100%. It even results in longer estrus duration, faster estrus onset, and higher estrus intensity. It was also found that the pregnancy rate for buffalo reached up to 80%. The results of this study indicate that the use of multivitamins in the estrus synchronization hormone protocol can increase the reproductive efficiency and productivity of buffalo livestock.

## List of Abbreviations

AI, Artificial insemination; BCS, Body condition score; GnRH, Gonadotropin-releasing hormone; ml, milliliter; PGF2α, Prostaglandin F2 α; T1, Treatment-1; T2, Treatment-2; T3, Treatment-3; SPSS, Statistical Package for the Social Sciences (Statistical program).
